# Supplementary material for: Resilient water infrastructure partnerships in institutionally complex systems face challenging supply and financial risk tradeoffs
Source: Nat Commun. 2024 Aug 27;15:7354. doi: 10.1038/s41467-024-51660-8 (PMC11350222; doi:10.1038/s41467-024-51660-8)
Supplement: Supplementary file 1 — Supplementary Information [file 41467_2024_51660_MOESM1_ESM.pdf]

Supplementary Information for "Resilient water infrastructure partnerships in institutionally complex systems face challenging supply and financial risk tradeoffs"

Hamilton, A.L.[1,2], Reed, P.M.[1], Gupta, R.S.[1], Zeff, H.B.[3,4], & G.W. Characklis[3,4]

[1]School of Civil and Environmental Engineering, Cornell University, Ithaca, NY, USA
[2]Confluency, Chicago, IL, USA
[3]Department of Environmental Sciences and Engineering, Gillings School of Global Public Health, University of North Carolina at Chapel Hill, Chapel Hill, NC, USA
[4]Center on Financial Risk in Environmental Systems, Gillings School of Global Public Health, UNC Institute for the Environment, University of North Carolina at Chapel Hill, Chapel Hill, NC, USA

Contents

1. Supplementary Figures S1-S18
2. Supplementary Tables S1-S4
3. Supplementary Notes S1-S3

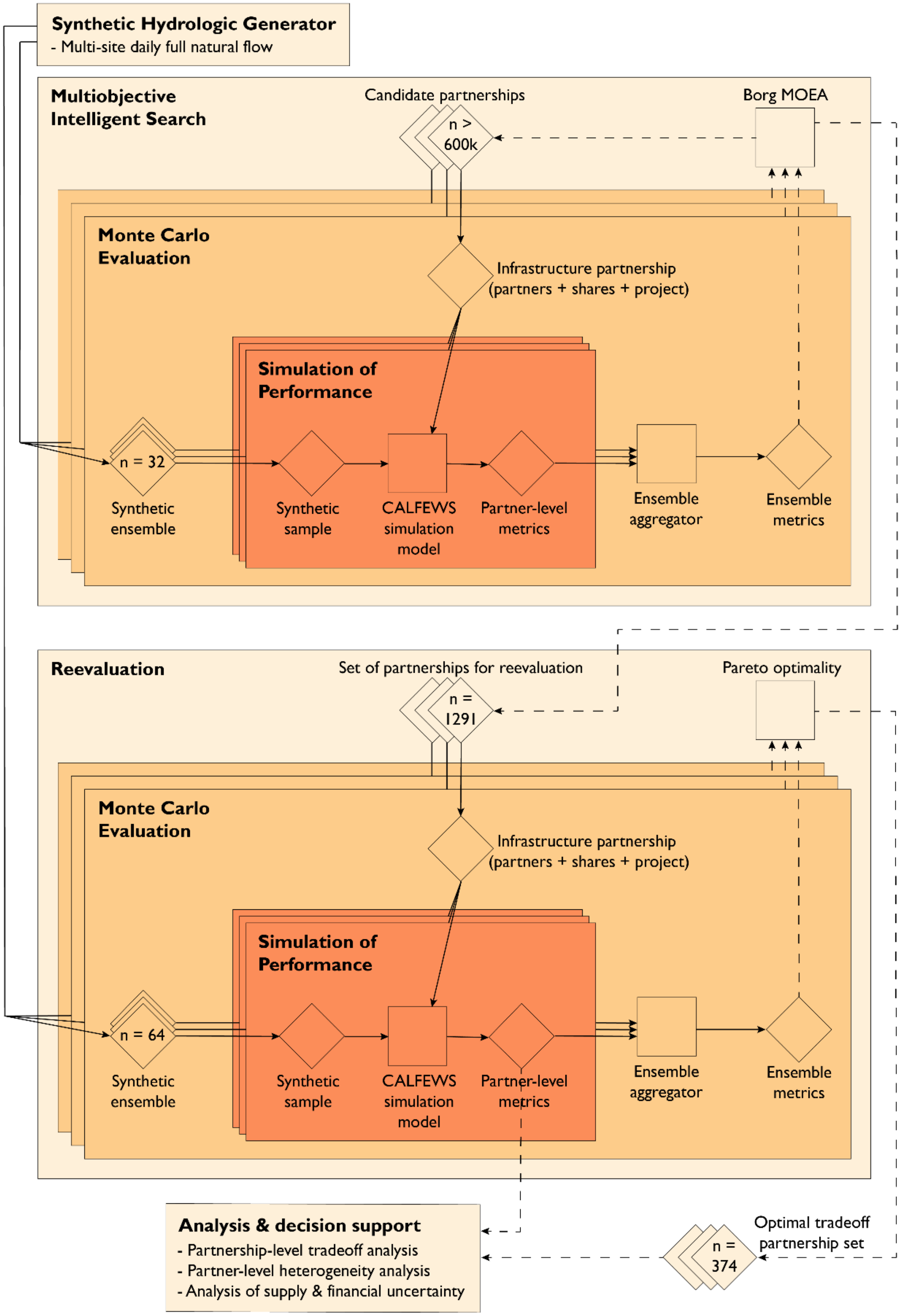

Supplementary Fig. S1: Overview of methodological framework.

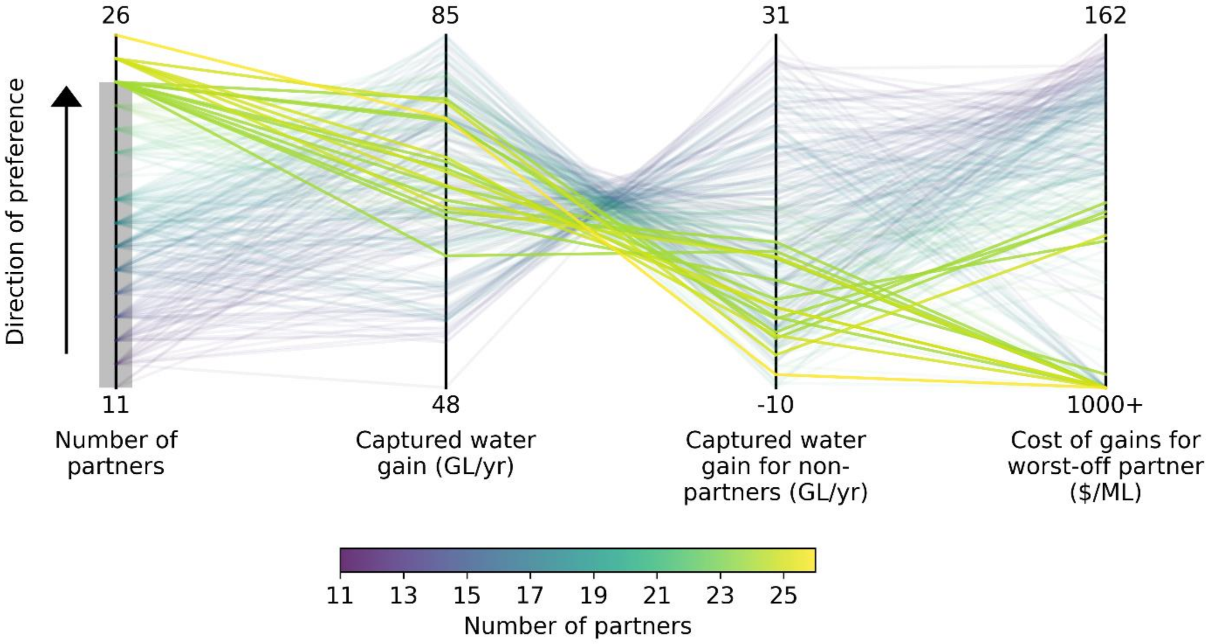

Supplementary Fig. S2: Parallel coordinate plot highlighting large partnerships. *All partnerships with fewer than 24 partners are screened out, as represented by the grey bar.*

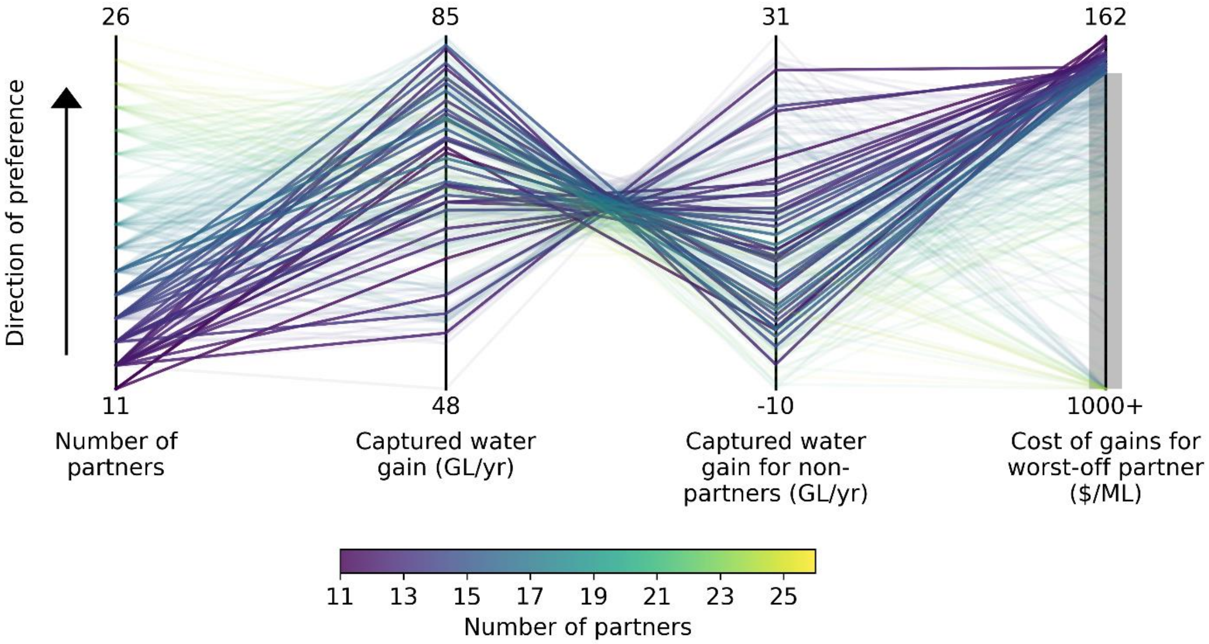

Supplementary Fig. S3: Parallel coordinate plot highlighting partnerships with low worst-partner cost of gains. *All partnerships that cost more than $200/ML for the worst-off partner in the 90th percentile of states-of-the-world are screened out, as represented by the grey bar.*

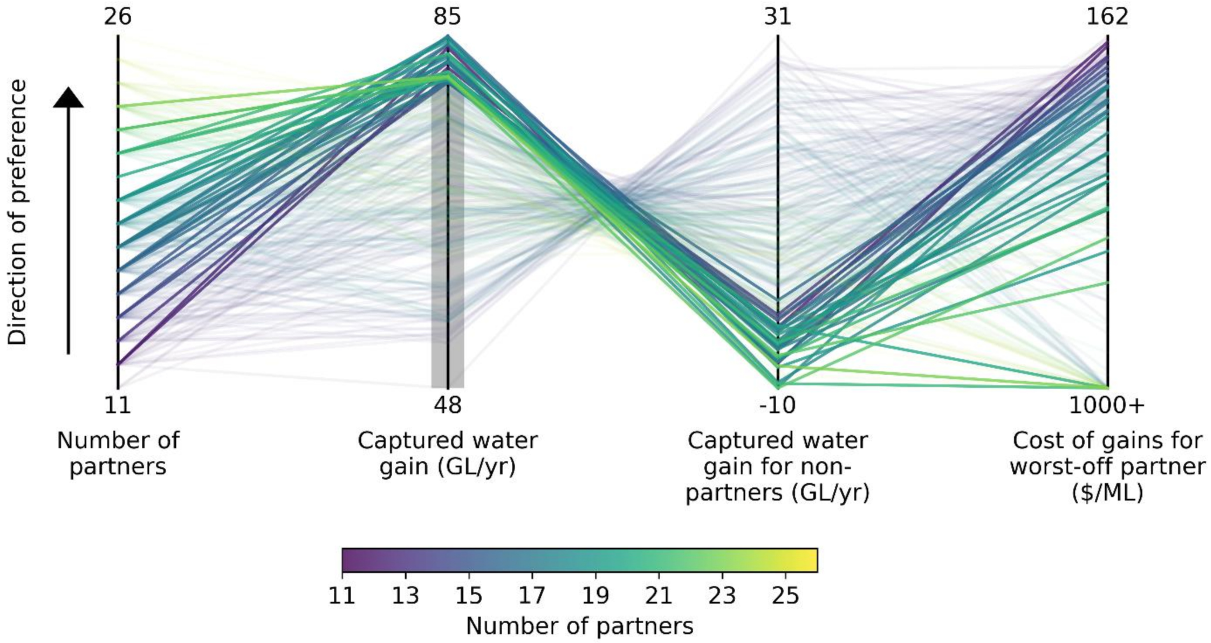

Supplementary Fig. S4: Parallel coordinate plot highlighting partnerships with large partner water supply gains. *All partnerships that generate less than 80 GL/year of captured water gains for partners are screened out, as represented by the grey bars.*

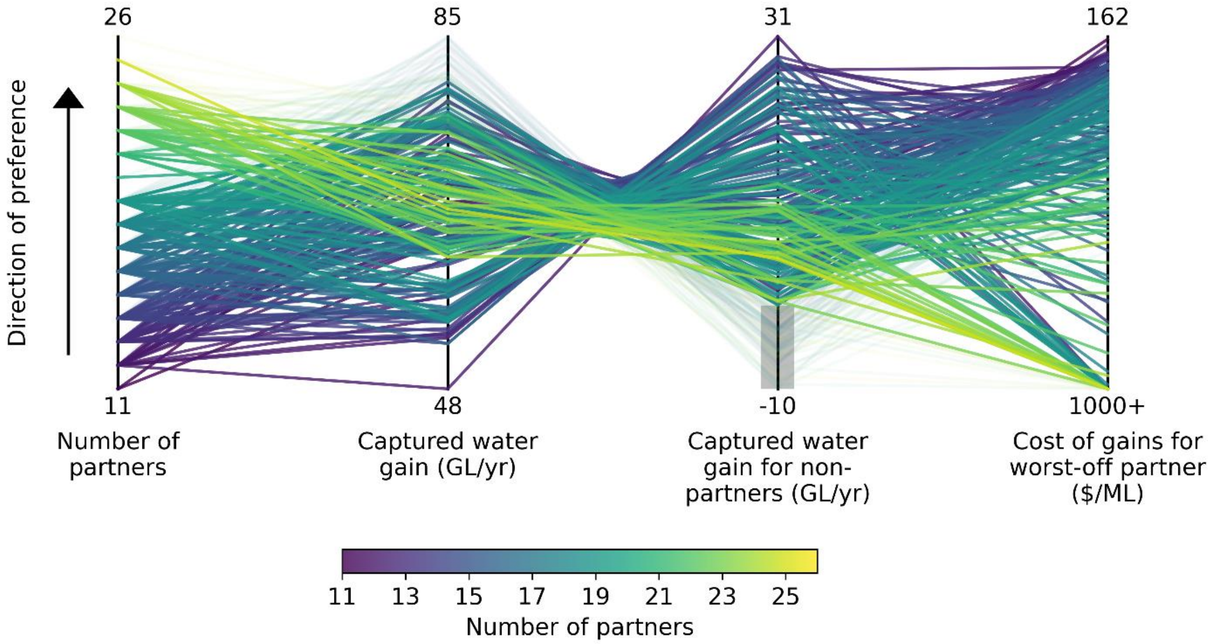

Supplementary Fig. S5: Parallel coordinate plot excluding partnerships with negative expected external impacts. *All partnerships that reduce the total average water deliveries to non-partners in the region are screened out, as represented by the grey bar.*

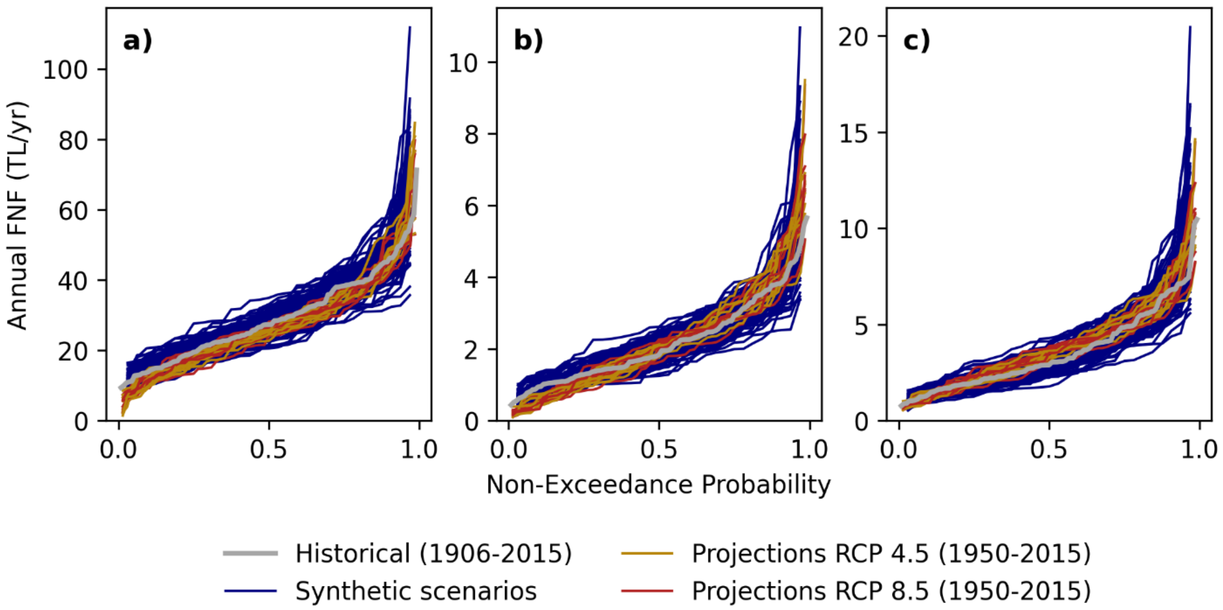

Supplementary Fig. S6: Comparison of full natural flow (FNF) distributions over the historical period. *Distributions are shown for* ***(a)*** *aggregated major surface water reservoirs north of Millerton Lake (Shasta, Oroville, New Bullards Bar, Folsom, New Melones, Don Pedro, McClure),* ***(b)*** *Millerton Lake,* ***(c)*** *and aggregated major surface water reservoirs south of Millerton Lake (Pine Flat, Kaweah, Success, Isabella). Each subfigure shows the non-exceedance curves for annual FNF. "Historical" refers to the 110-year historical full natural flow reanalysis dataset. "Synthetic" refers to the 100 different 30-year synthetic flow scenarios used during the multiobjective intelligent search and reevaluation steps. "Projections 4.5" and "Projections 8.5" refer to the 1950-2015 results from 10 downscaled CMIP5 models using RCP 4.5 and RCP 8.5.*

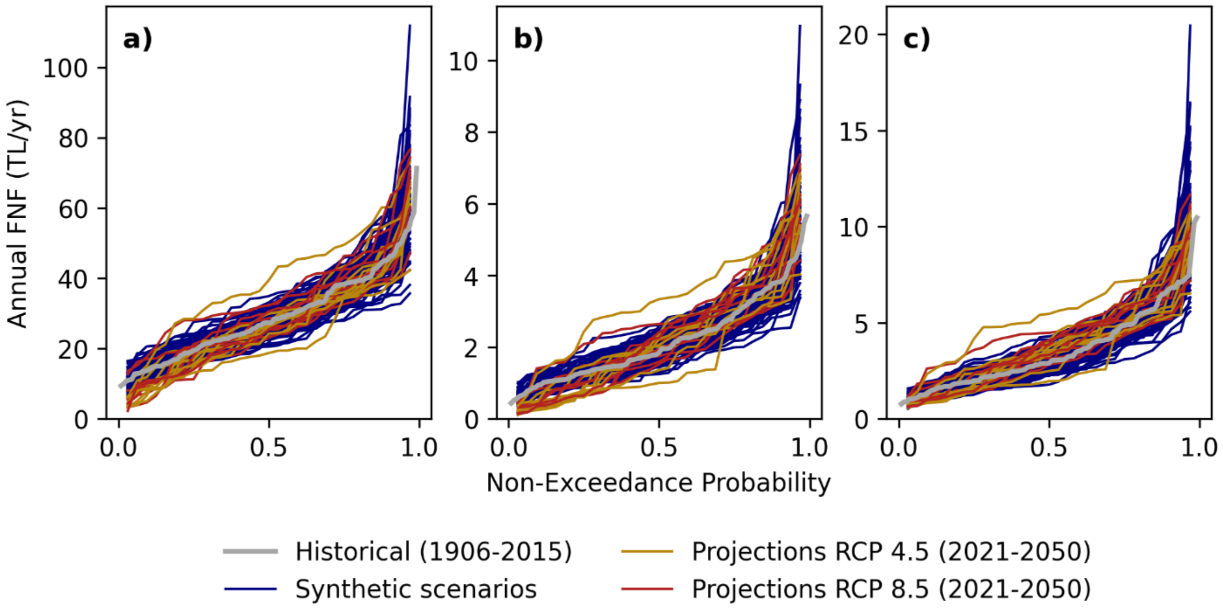

Supplementary Fig. S7: Comparison of full natural flow (FNF) distributions over the 2021-2050 period. *Distributions are shown for* ***(a)*** *aggregated major surface water reservoirs north of Millerton Lake (Shasta, Oroville, New Bullards Bar, Folsom, New Melones, Don Pedro, McClure),* ***(b)*** *Millerton Lake,* ***(c)*** *and aggregated major surface water reservoirs south of Millerton Lake (Pine Flat, Kaweah, Success, Isabella). Each subfigure shows the non-exceedance curves for annual FNF. "Historical" refers to the 110-year historical full natural flow reanalysis dataset. "Synthetic" refers to the 100 different 30-year synthetic flow scenarios used during the multiobjective intelligent search and reevaluation steps. "Projections 4.5" and "Projections 8.5" refer to the 2021-2050 results from 10 downscaled CMIP5 models using RCP 4.5 and RCP 8.5.*

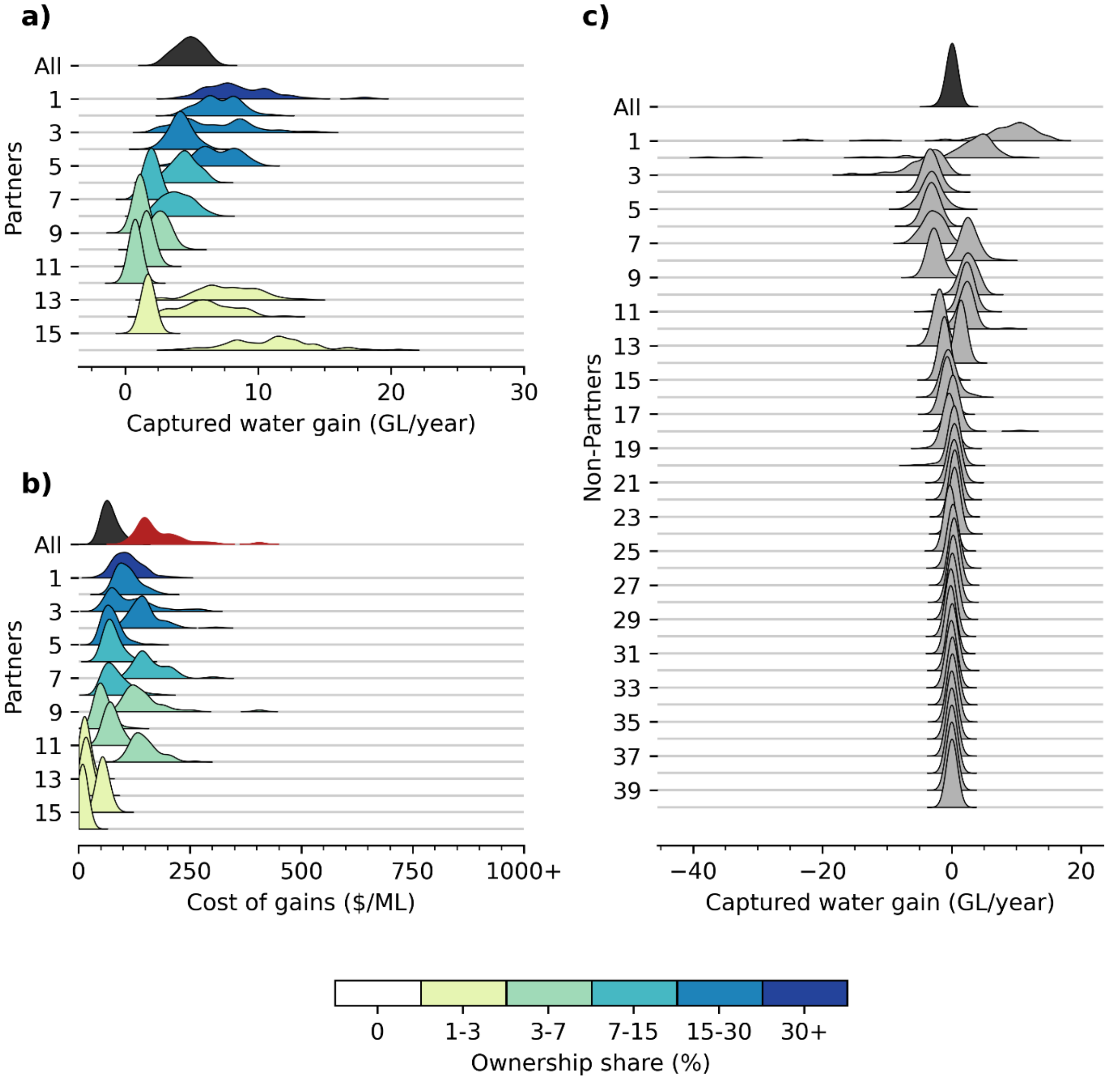

Supplementary Fig. S8: Disaggregation of performance for the Compromise Partnership. (a) *Captured water gain for each of the 16 water provider partners, sorted and colored by ownership share. Each distribution shows a Kernel Density Estimate across 64 sampled hydrologic scenarios. The black distribution ("All") shows the average performance across all partners.* ***(b)*** *Same as (a), but for the cost of gains. The red distribution ("All") shows the maximum cost across all partners in each scenario.* ***(c)*** *Same as (a), but showing the captured water gain for 26 non-partner water providers, ordered by their expected absolute deviation from 0.*

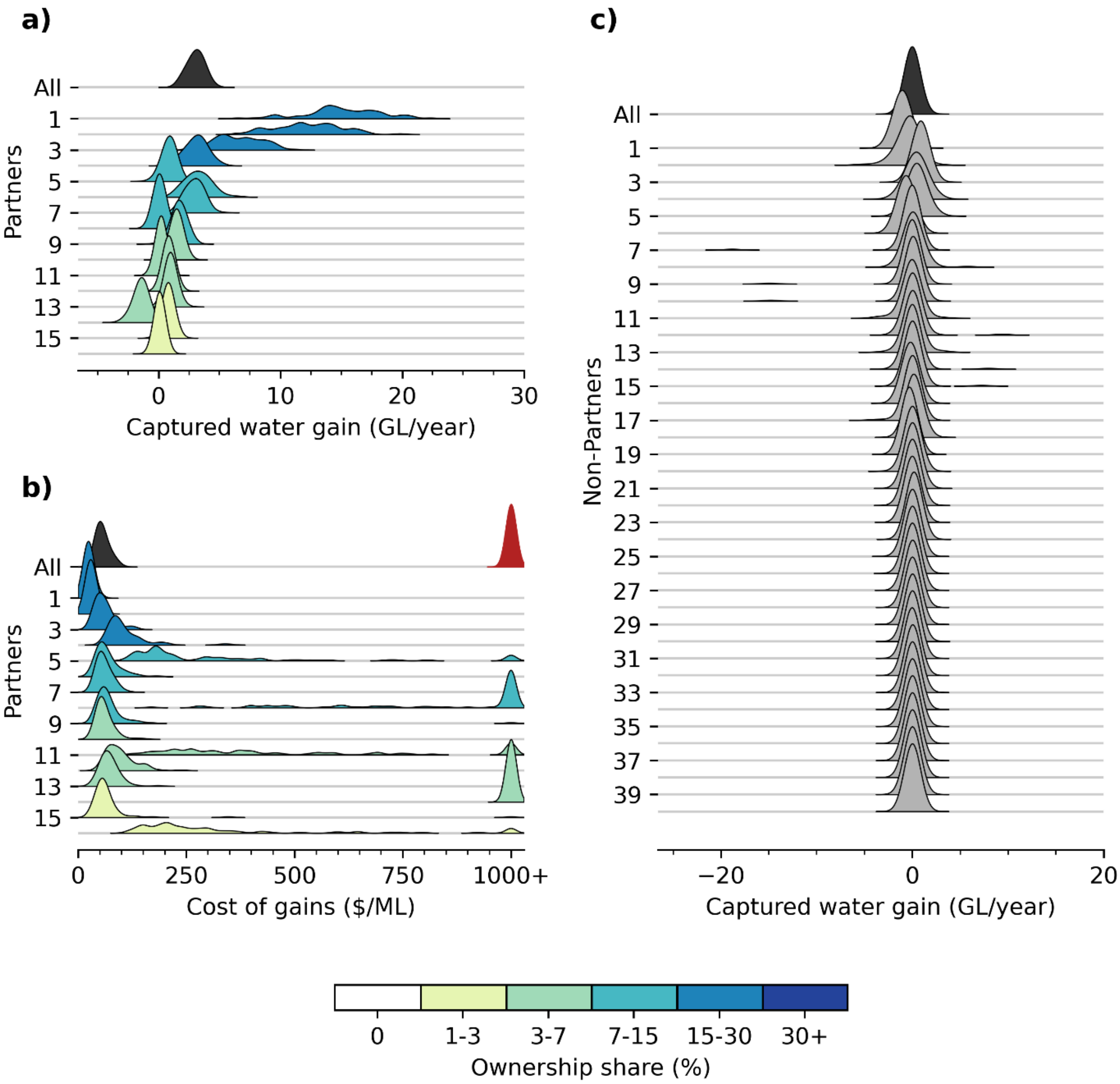

***Supplementary Fig. S9: Disaggregation of performance for the Status Quo Partnership. (a)** Captured water gain for each of 16 water provider partners, sorted and colored by ownership share. Each distribution shows a Kernel Density Estimate across 64 sampled hydrologic scenarios. The black distribution ("All") shows the average performance across all partners. **(b)** Same as (a), but for the cost of gains. The red distribution ("All") shows the maximum cost across all partners in each scenario. The cost of gains is capped at $1000/ML. **(c)** Same as (a), but showing the captured water gain for 26 non-partner water providers, ordered by their expected absolute deviation from 0.*

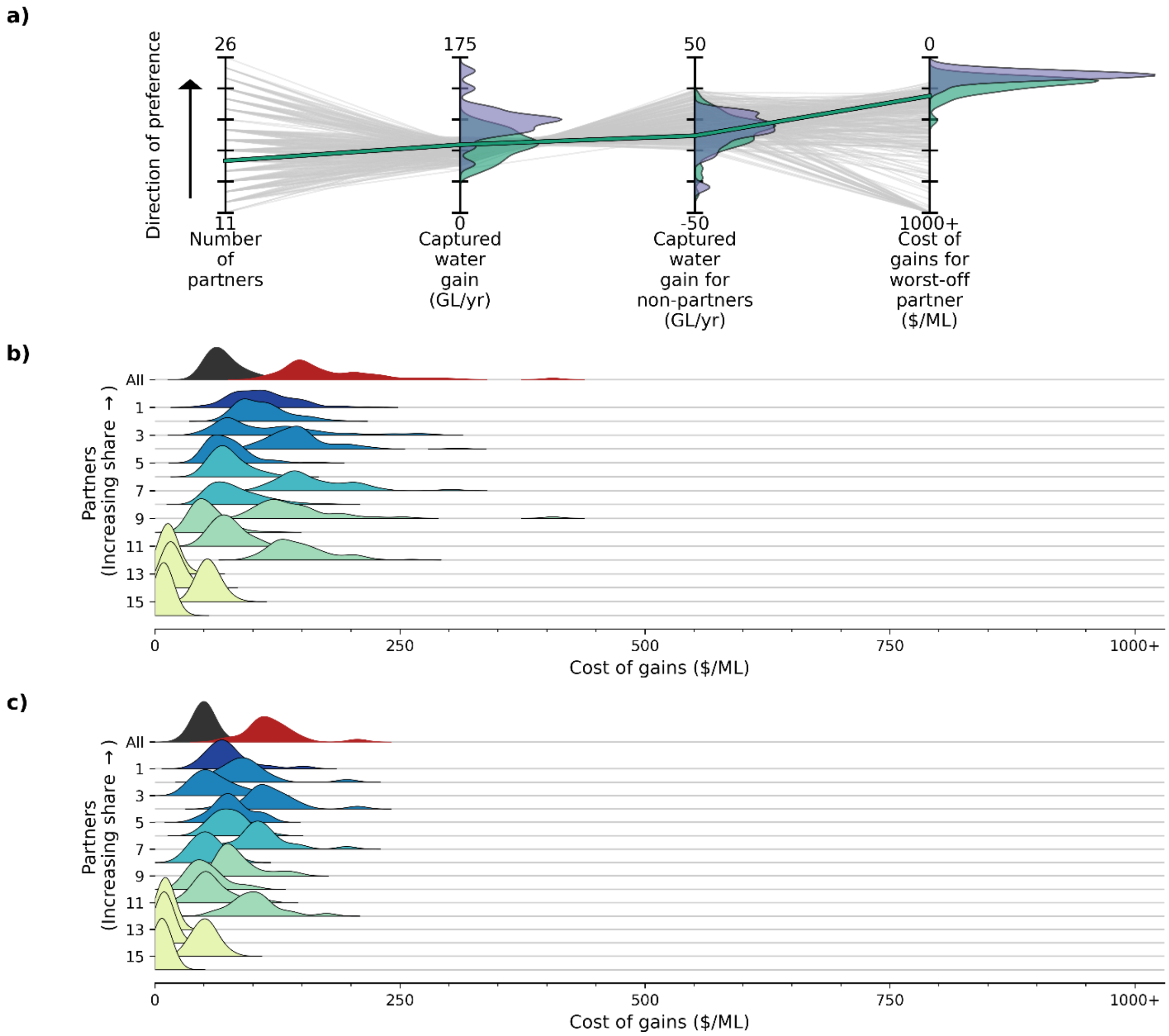

Supplementary Fig. S10: Uncertain performance of Compromise Partnership in synthetic scenarios vs. CMIP5 scenarios. (a) *Parallel coordinate plot showing partnership performance across four conflicting objectives. Each gray line represents the performance of a different optimal tradeoff partnership, aggregated across the 79 synthetic 30-year daily hydrologic sequences. The green line represents the aggregated performance of the Compromise Partnership across the synthetic scenarios, while the green shaded areas show the probability distribution of single-synthetic-scenario performances for the Compromise Partnership. For comparison, the blue shaded area represents the probability distribution of single-CMIP5-scenario performances for the Compromise Partnership.* ***(b)*** *Heterogeneity of partner-level performance for the Compromise Partnership across the synthetic scenarios. Each shaded area labeled 1-16 represents the distribution of costs of gains for a different water provider partner across the 79 sampled 30-year daily hydrologic sequences. The partners are sorted and colored by ownership share. The distributions labeled "All" represent the partnership-level aggregated costs (black) and the costs for the worst-off partner across alternative scenarios.* ***(c)*** *Same as (b), but for the CMIP5 scenarios.*

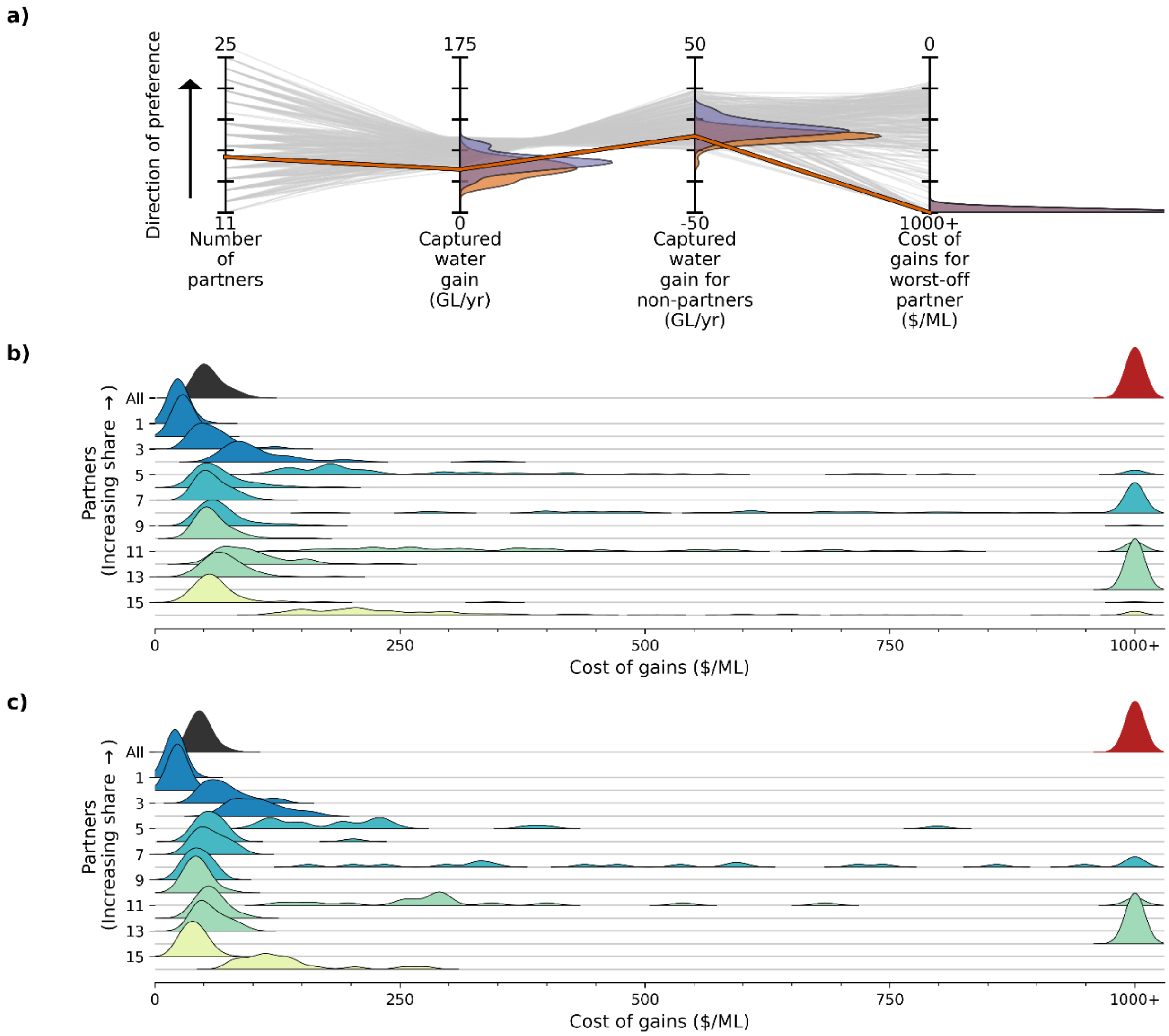

Supplementary Fig. S11: Uncertain performance of Status Quo Partnership in synthetic scenarios vs. CMIP5 scenarios. (a) *Parallel coordinate plot showing partnership performance across four conflicting objectives. Each gray line represents the performance of a different optimal tradeoff partnership, aggregated across the 79 synthetic 30-year daily hydrologic sequences. The orange line represents the aggregated performance of the Status Quo Partnership across the synthetic scenarios, while the orange shaded areas show the probability distribution of single-synthetic-scenario performances for the Status Quo Partnership. For comparison, the blue shaded area represents the probability distribution of single-CMIP5-scenario performances for the Status Quo Partnership.* ***(b)*** *Heterogeneity of partner-level performance for the Status Quo Partnership across the synthetic scenarios. Each shaded area labeled 1-16 represents the distribution of costs of gains for a different water provider partner across the 79 sampled 30-year daily hydrologic sequences. The partners are sorted and colored by ownership share. The distributions labeled "All" represent the partnership-level aggregated costs (black) and the costs for the worst-off partner across alternative scenarios.* ***(c)*** *Same as (b), but for the CMIP5 scenarios.*

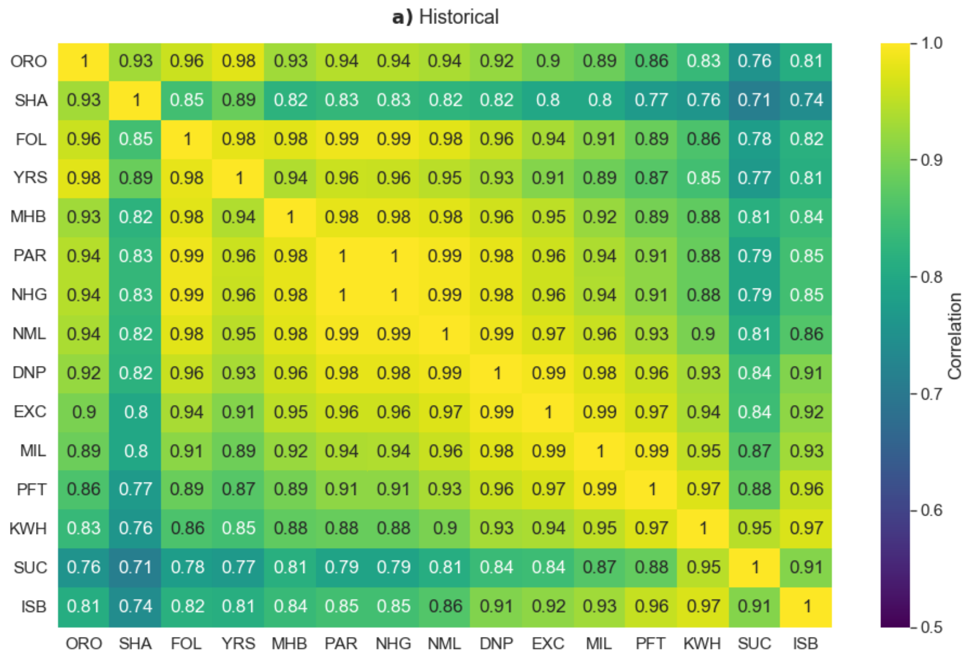

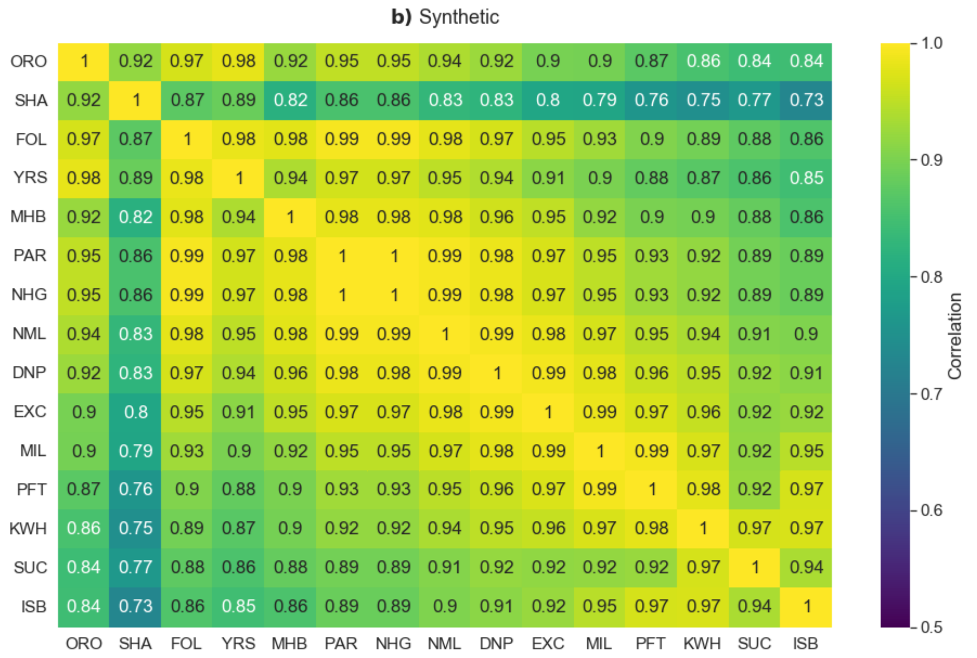

Supplementary Fig. S12*: *Annual correlations across sites *calculated from **(a)** the historical 110-year record and **(b)** a sampled 110-year synthetic record from the synthetic hydrologic generator.*

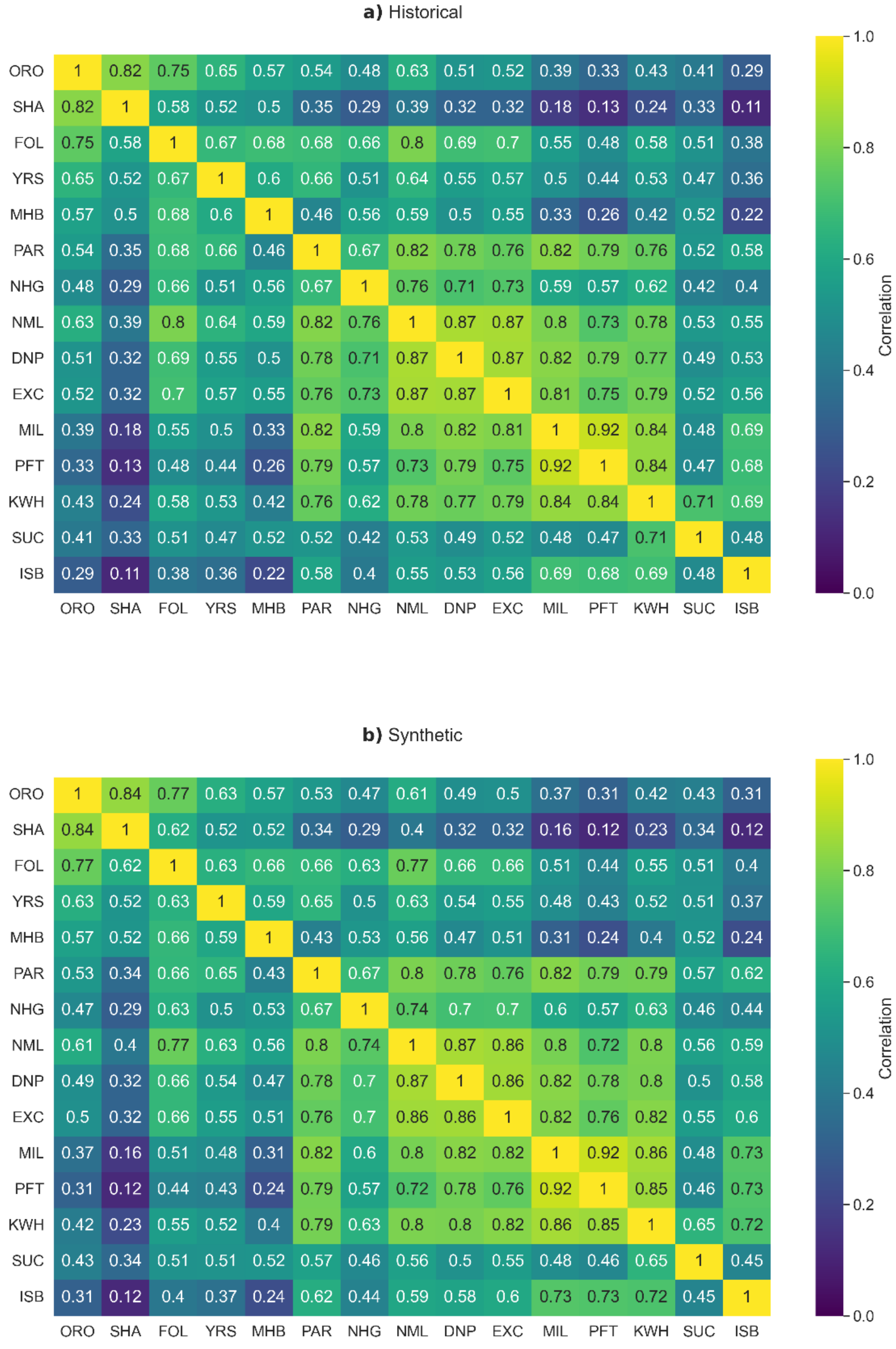

Supplementary Fig. S13: Daily correlations across sites *calculated from* ***(a)*** *the historical 110-year record and* ***(b)*** *a sampled 110-year synthetic record from the synthetic hydrologic generator.*

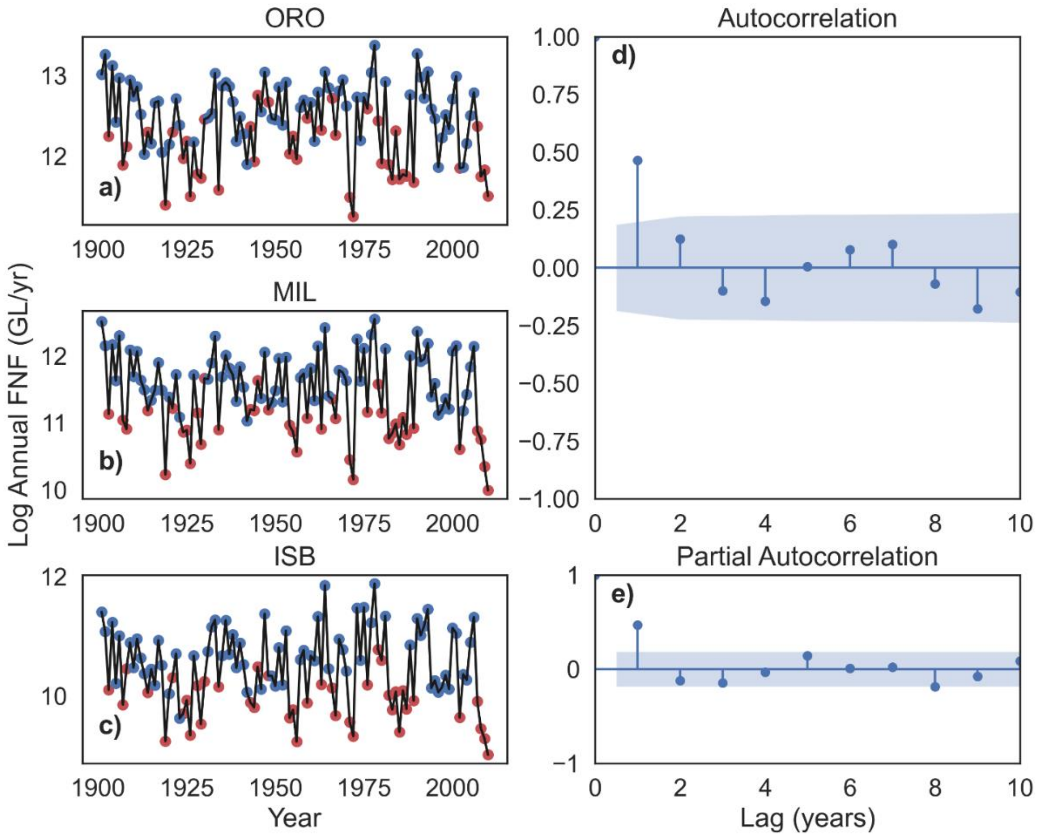

Supplementary Fig. S14: Temporal autocorrelation for wet and dry states. *Classification of annual record into wet and dry states for full natural flow (FNF) at* ***(a)*** *Oroville,* ***(b)*** *Millerton, and* ***(c)*** *Isabella. Since locations share a common transition matrix, the state assigned for each location is the same for each year. The Markov property of the shared hidden state vector is demonstrated through the* ***(d)*** *autocorrelation function and* ***(e)*** *partial autocorrelation function.*

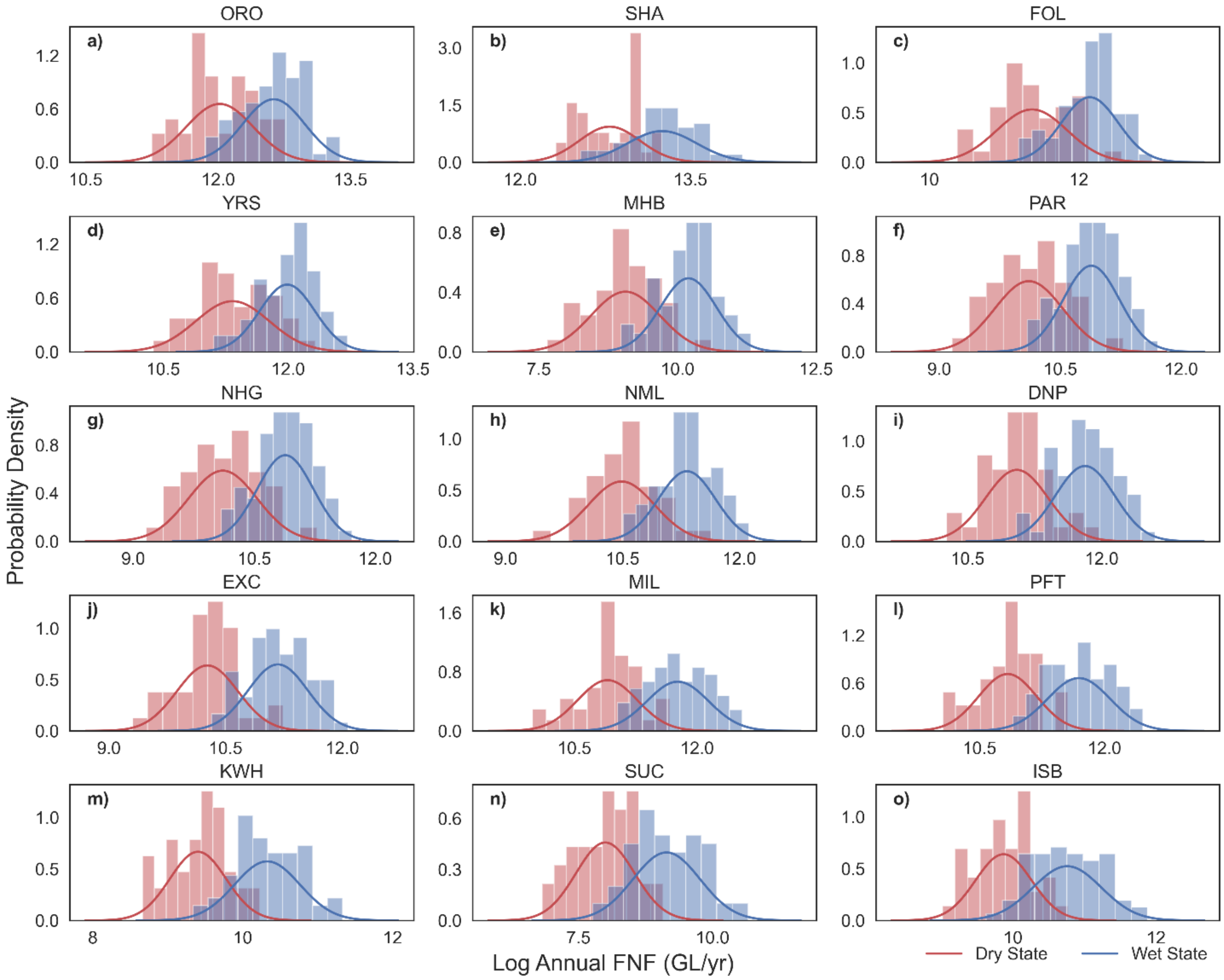

***Supplementary Fig. S15: Fitted distributions of wet and dry state** log-space annual full natural flows for each site: Oroville (a), Shasta (b), Folsom (c), New Bullards Bar (d), Michigan Bar (e), Pardee (f), New Hogan (g), New Melones (h), Don Pedro (i), New Exchequer (j), Millerton (k), Pineflat (l), Kaweah (m), Success (n), and Isabella (o).*

lso

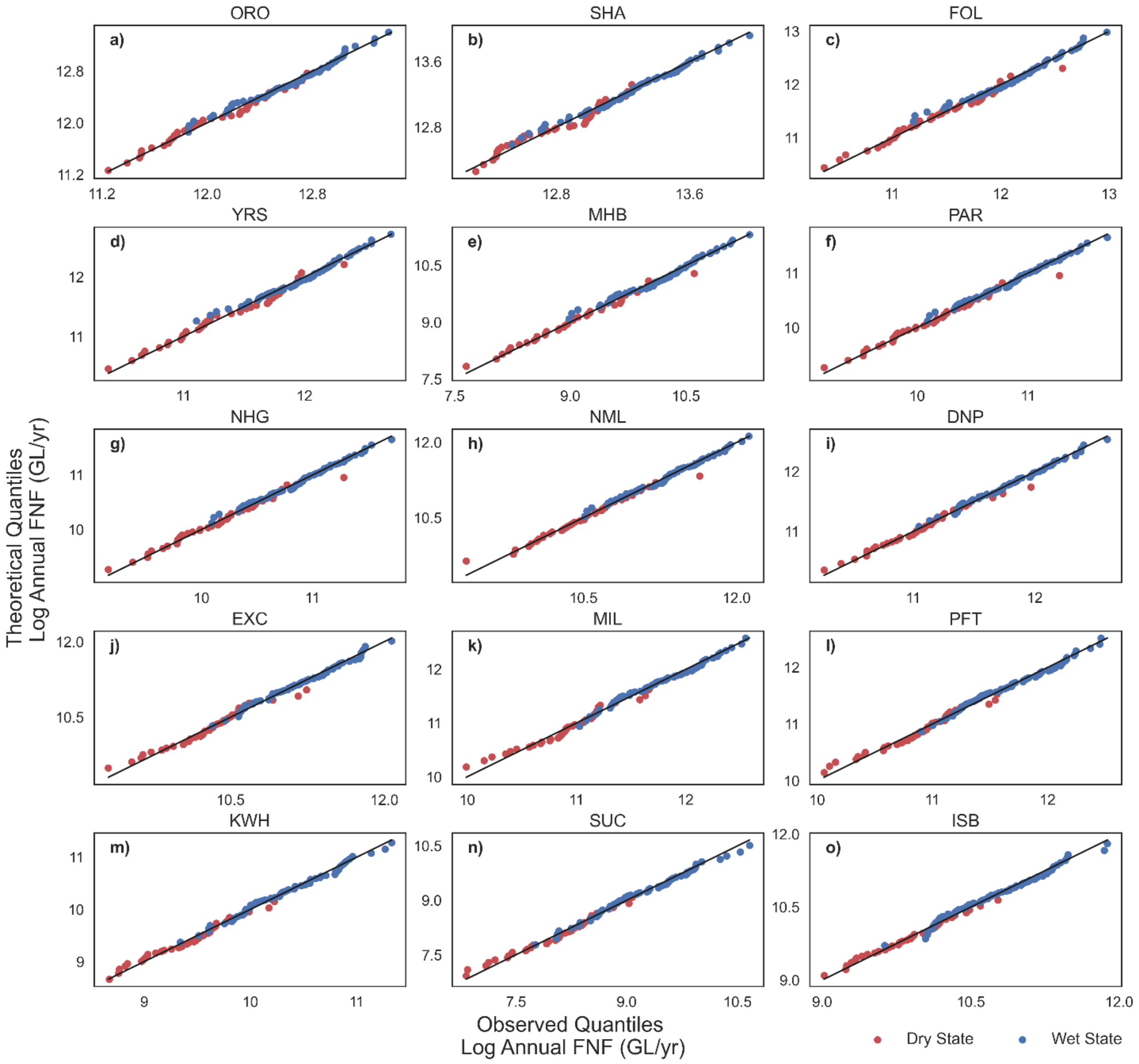

***Supplementary Fig. S16: QQ plots for fitted distributions of wet and dry state** log-space annual full natural flows for each site: Oroville (a), Shasta (b), Folsom (c), New Bullards Bar (d), Michigan Bar (e), Pardee (f), New Hogan (g), New Melones (h), Don Pedro (i), New Exchequer (j), Millerton (k), Pineflat (l), Kaweah (m), Success (n), and Isabella (o).*

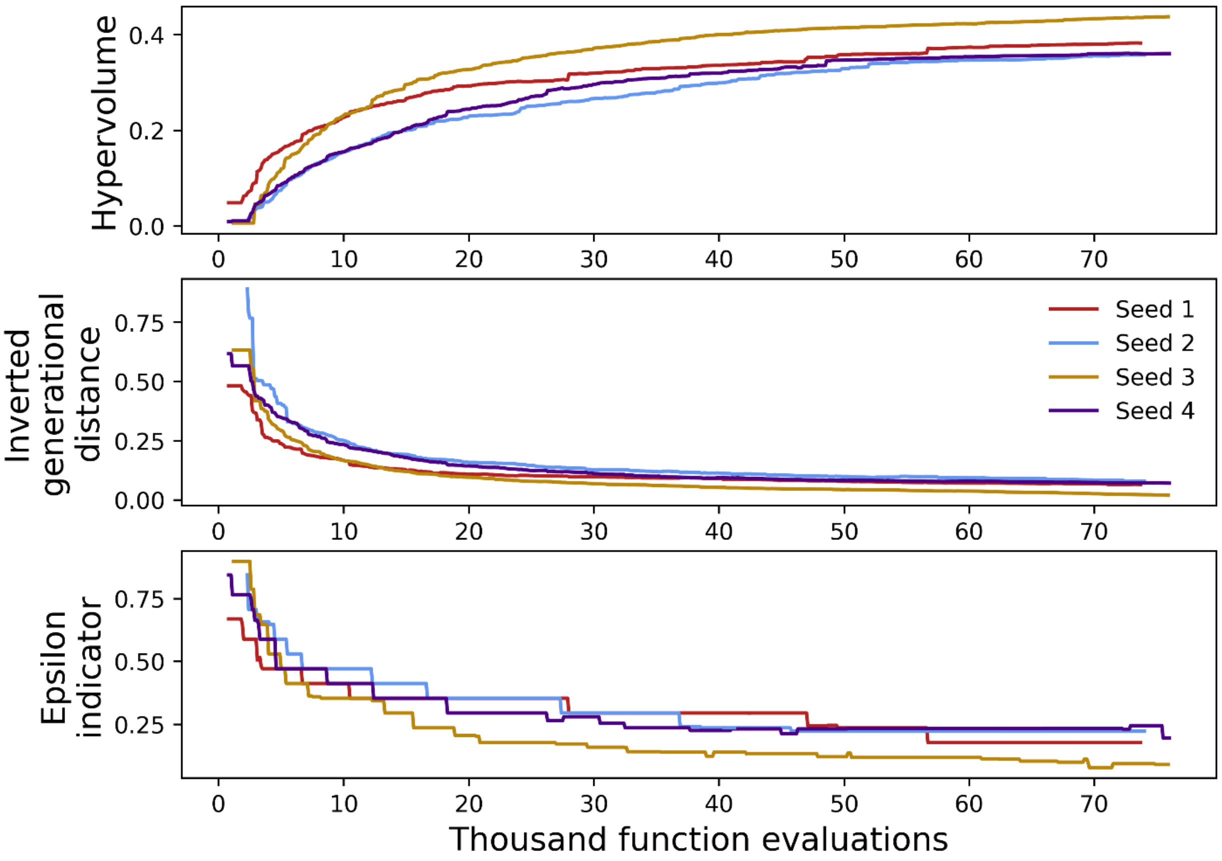

Supplementary Fig. S17: Progress of multiobjective intelligent search according to three metrics. *The preferred direction of progress is up for hypervolume (top) and down for inverted generational distance (middle) and epsilon indicator (bottom). Each of the four random seed replicates of the Borg MOEA are shown.*

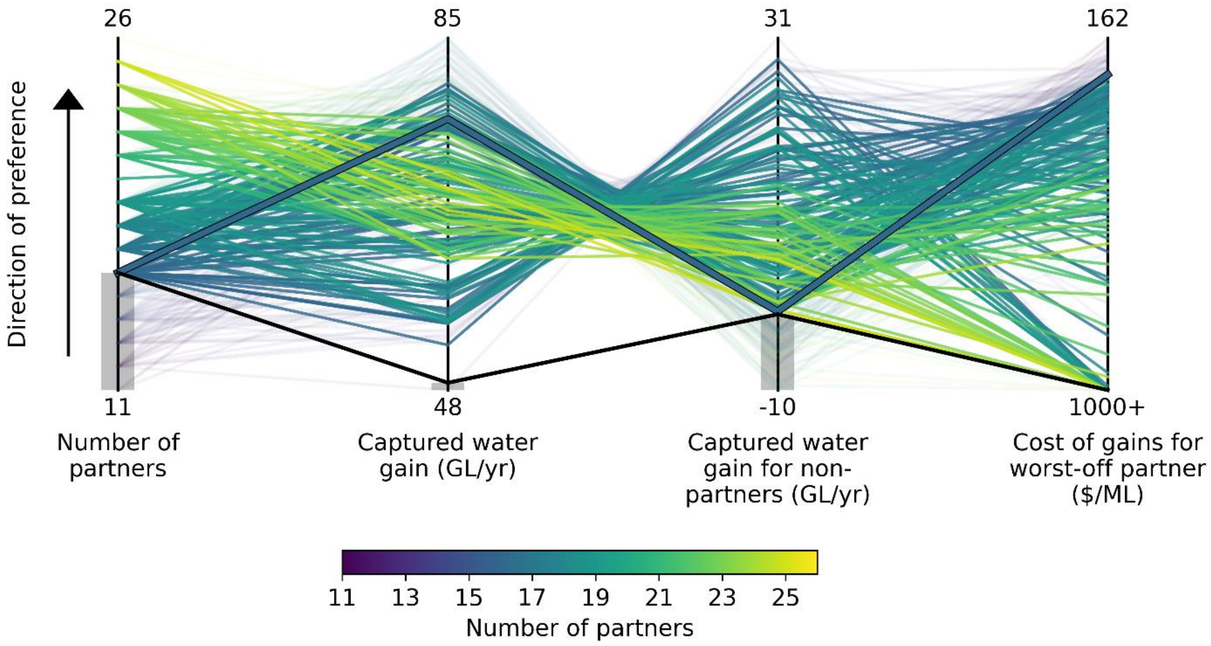

Supplementary Fig. S18: Parallel coordinate plot highlighting partnerships that outperform the Status Quo Partnership*. All partnerships that perform worse than the Status Quo Partnership (black line) on at least one objective are screened out, as represented by the grey bars. The Compromise Partnership (bolded blue line with black outline) is selected from among the highlighted partnerships as the one minimizing the worst-partner cost of gains metric.*

***Supplementary Table S1**: The log space dry and wet state means associated with each site in the Hidden Markov Model: Oroville (a), Shasta (b), Folsom (c), New Bullards Bar (d), Michigan Bar (e), Pardee (f), New Hogan (g), New Melones (h), Don Pedro (i), New Exchequer (j), Millerton (k), Pineflat (l), Kaweah (m), Success (n), and Isabella (o).*

	ORO	SHA	FOL	YRS	MHB	PAR	NHG	NML	DNP	EXC	MIL	PFT	KWH	SUC	ISB
Dry	14.8	15.6	14.2	14.1	11.9	12.9	12.9	13.3	13.8	13.1	13.7	13.6	12.2	10.8	12.7
Wet	15.4	16.0	14.9	14.8	13.0	13.7	13.7	14.1	14.6	14.0	14.6	14.5	13.1	11.9	13.6

***Supplementary Table S2**: The log space dry and wet state variances associated with each site in the Hidden Markov Model: Oroville (a), Shasta (b), Folsom (c), New Bullards Bar (d), Michigan Bar (e), Pardee (f), New Hogan (g), New Melones (h), Don Pedro (i), New Exchequer (j), Millerton (k), Pineflat (l), Kaweah (m), Success (n), and Isabella (o).*

	ORO	SHA	FOL	YRS	MHB	PAR	NHG	NML	DNP	EXC	MIL	PFT	KWH	SUC	ISB
Dry	0.14	0.07	0.22	0.20	0.38	0.18	0.18	0.18	0.12	0.15	0.13	0.12	0.14	0.30	0.15
Wet	0.12	0.09	0.14	0.11	0.25	0.12	0.12	0.13	0.11	0.15	0.14	0.14	0.19	0.39	0.22

***Supplementary Table S3**: The wet and dry state transition probability matrix that is shared across sites in the Hidden Markov Model.*

	Dry	*Wet*
Dry	0.490	0.510
Wet	0.302	0.697

***Supplementary Table S4**: Definitional parameters for the four performance metrics.*

	Number of partners	*Captured water gain*	*Captured water gain for non-partners*	*Cost of gains for worst-off partner*
Units	None	GL/yr	GL/yr	$/ML
Water provider aggregation method	Sum	Sum	Sum	Max (Worst-case)
Hydrologic scenario aggregation method	None	Mean	Mean	90th percentile
Direction of preference for optimization	Max	Max	Max	Min
Epsilon-dominance parameter	0.999	2	2	5

Supplementary Note S1: Additional detail on hydrologic scenarios

The 110-year multisite historical full natural flow (FNF) reanalysis dataset described in Zeff et al. (2021)[1] is used for training and verification of the synthetic hydrologic scenario generator. Supplementary Fig. S12a demonstrates that the historical FNF at the 15 locations are highly correlated at an annual scale, which suggests that a unified model that preserves this spatial correlation is necessary. The overall multi-site HMM model is a mixture model defined by a transition probability matrix between the hidden states (wet and dry) along with a separate Gaussian multivariate distribution for the 15 sites within each of the two states. The wet and dry state distributions have respective mean vectors of length 15 and covariance matrices of size 15x15 that captures the joint variability across the sites. Because the inflows across the sites are highly correlated it is assumed they share a common state in any given year. Thus, a single 2x2 transition matrix dictates the likelihood of persisting and transitioning between a wet and dry state in a given year. Though this study fits the HMM model under stationary conditions, in future work the parameters of the HMM can be systematically adjusted to generate flows that are representative of plausible nonstationary future climate conditions, such as increased drought persistence, changes to the mean wet/dry states, and spatially compounding drought or flood behavior across the region.

We fit the multi-site Gaussian HMM to log annual FNF across the length of the 110-year record. The parameter estimation is performed using Expectation-Maximization with Python's *hmmlearn* package[2]. The values for all parameters are shown in Supplementary Tables S1-S3 and site-level component distributions are visualized in Supplementary Fig. S15. State identification of wet and dry years in the historical record, shown in Supplementary Fig. S14a-c, is performed using the Viterbi algorithm with the same Python library. There is considerable persistence in the system that is captured by the model and demonstrated in the transition matrix in Supplementary Table S1. The appropriateness of the Markov property assumption embedded in the HMM model is demonstrated by the autocorrelation and partial autocorrelation plots which show that only the single-year lag provides statistically significant information in the inferred hidden state sequence for the historical record (Supplementary Fig. S14d-e).

To create new synthetic sequences, we sample from the model to first generate a series of hidden states at the annual timescale. Then we sample log-space synthetic FNF at each site from the state-specific multivariate Gaussian distributions. The log-space annual FNF are converted to real space and temporally downscaled to daily FNF using the proportional scaling method introduced by Nowak et al. (2010)[3]. First, for each synthetic year, the historical year with the nearest annual total FNF summed across all sites is selected as a reference. Then each site’s synthetic FNF is disaggregated to daily FNF based on the daily flow proportions in the reference year record for that site. The maintenance of the daily and annual correlations by the synthetic generator is shown in Supplementary Figs. S12-S13. An analysis of the goodness of fit of the model at each site is shown in Supplementary Fig. S16.

Supplementary Fig. S6 shows that the 100 synthetically sampled 30-year streamflow scenarios used in this study follow a similar annual non-exceedance probability curve compared to the 1906-2015 historical record which was used to train the synthetic generator. At the same time, the ensemble of synthetic scenarios is found to substantially widen the envelope of extreme high- and low-flows compared to the historical record. This is critical for accurately characterizing risk in long-lived infrastructure investments due to the critical impact of hydroclimatic internal variability in regions like California. The first 21 of these flow sequences are used in the optimization stage of the modeling framework and the other 79 flow sequences are used in the reevaluation phase.

In order to compare our synthetically generated streamflow ensemble to the streamflows that could be experienced under anthropogenic climate change over a 30-year investment horizon, we also consider an ensemble of downscaled climate change scenarios from the Climate Model Intercomparison Project Phase 5 (CMIP5)[4]. Brekke et al. (2013) previously released a suite of hydrologic scenarios for California generated by using downscaled CMIP5 simulations to drive the Variable Infiltration Capacity (VIC) hydrologic model[5,6]. Cohen et al. (2020) then aggregated and organized these results to get basin streamflows to drive a reservoir operations model for California[7]. We utilize a subset of streamflow scenarios from Cohen et al. (2020) for 10 CMIP5 models: CCSM4[8], CNRM-CM5.1[9], CSIRO-Mk3.6.0[10], GFDL-CM3[11], GFDL-ESM2M[12], HADGEM2-CC[13], HADGEM2-ES[13], INMCM4[14], IPSL-CM5A-MR[15], and MIROC5[16]. For each model, we use both RCP 4.5 and 8.5, for a total of 20 combinations.

Compared to the synthetically generated streamflow scenarios, the 20 downscaled CMIP5 scenarios show more limited range of variability and extremes over the 1950-2015 period (Supplementary Fig. S6). They also appear to show a consistent low bias in the driest years over the historical period. Over the 2021-2050 period, the downscaled CMIP5 scenarios are found to display a wider envelope of extreme annual behavior compared to the historical period (Supplementary Fig. S7). The envelope of extremes in this case is more similar to the synthetically generated scenarios. This shows that although the synthetic generator is trained on the historical record, it nonetheless is able to generate scenarios with similarly extreme behavior as found in the CMIP5 scenarios which explicitly account for anthropogenic climate change.

Supplementary Note S2: Additional detail on performance metrics

Each candidate infrastructure partnership is evaluated according to four decision-relevant metrics. Various characteristics of the four metrics are summarized in Supplementary Table S4.

The first metric, M_P^1, is the number of water provider partners participating in partnership P:

$$M_P^1 = \sum_{w \in P} 1$$

where the summation is over the subset of water providers w that are partners in partnership P. The number of partners is fixed across hydrologic scenarios, so no aggregation method is needed. This metric is maximized in the multiobjective optimization step.

The second metric is the captured water gain, defined as the expected increase in total surface water deliveries to project partners:

$$M_P^2 = \frac{1}{N_h * 30} \sum_h \sum_{w \in P} (\mathrm{CW}[w, h, P] - \mathrm{CW}[w, h, P_0])$$

where the outer summation is over the $N_h = 32$ sampled 30-year hydrologic scenarios h used in the multiobjective optimization or the $N_h = 64$ sampled scenarios used in the reevaluation step. The inner summation is over the water provider partners w within partnership P. The summand calculates the captured water CW delivered in hydrologic scenario h to water provider w in partnership P, minus the captured water delivered to the same partner in the same hydrologic scenario in the counterfactual

baseline case P_0 where the infrastructure partnership does not exist. The division by $N_h * 30$ converts the metric into an expected annual value (GL/yr). This metric is maximized in the multiobjective optimization step.

The third metric is the captured water gain for non-partner water providers in the region:

$$M_P^3 = \frac{1}{N_h * 30} \sum_h \sum_{w \notin P} (\mathrm{CW}[w,h,P] - \mathrm{CW}[w,h,P_0])$$

This is identical to M_P^2, except that the inner summation is over non-partners rather than partners. This metric is positive when total deliveries to non-partners are higher with the infrastructure partnership P than without it, and negative when total deliveries to non-partners are lower with the infrastructure partnership P than without it. This metric is maximized in the multiobjective optimization step.

The fourth and final metric is the cost of gains for the worst-off partner, defined as the annual debt payment divided by captured water gain for the worst-off partner in a partnership:

$$M_P^4 = Q90_h \left(\max_{w \in P} \left(\frac{\mathrm{AD}[P] * \mathrm{OS}[w,P]/100}{(\mathrm{CW}[w,h,P] - \mathrm{CW}[w,h,P_0])/30} \right) \right)$$

where the numerator of the inner argument represents the annual debt payment obligation for water provider partner w within partnership P given the total partnership annual debt payment $\mathrm{AD}[P]$ and partner w's ownership share $\mathrm{OS}[w,P]$ (in %). The denominator of the inner argument is the disaggregated annual captured water gain for partner w within partnership P in hydrologic scenario h. Taken as a whole, the inner argument represents the disaggregated cost of gains for a single partner in a single hydrologic scenario. The aggregate metric M_P^4 is calculated by first finding the cost of gain for the worst-off partner in each scenario (i.e., the maximum across partners), and then finding the 90th percentile of this quantity across sampled hydrologic scenarios. This metric is minimized in the multiobjective optimization step. The 90th percentile aggregation across hydrologic scenarios and the worst-case aggregation across partners are selected to favor partnerships that can provide robust affordable water supply benefits to all participating partners across a wide range of plausible hydrologic conditions. This problem formulation draws from previous efforts to define robust multiobjective problem formulations in noisy water resource simulation-optimization contexts[17–19].

Metrics M_P^2 to M_P^4 have been aggregated across multiple sampled hydrologic scenarios h and multiple water providers w. This is necessary to reduce performance to scalar values for optimization purposes. However, it is also informative to consider the broader ensembles of disaggregated performance values. Figs. 4b and 5b show the distributions of single-scenario performance for each metric for two different partnerships. These distributions do aggregate across water providers w following the equations above, but forgo the aggregation over hydrologic scenarios h. Further, Figs. 4c and 5c and Supplementary Figs. S8 and S9 show the distributions of single-scenario performance for individual water providers (i.e., forgoing both aggregation steps).

Supplementary Note S3: Additional detail on decision vector formulation

Within the multiobjective intelligent search process based on simulation-optimization, it is necessary to

define an explicit mapping from the numeric decision vector that is optimized to the instantiated infrastructure partnership that is simulated. This mapping is not unique for many applications, and alternative decision vector formulations can often exhibit widely varying learning speeds and capabilities within the heuristic search[20]. This is especially true in combinatorically-challenging portfolio-style problems, like the present study, where the goal is to select appropriate weights for a small subset of a larger set of options to include in the portfolio, while setting the rest of the options' weights to zero[21].

After comparing three alternative formulations in preliminary trials, the following formulation was selected based on its ability to facilitate efficient learning for the multiobjective evolutionary algorithm. The formulation has a decision vector with 85 elements defining partnership *P*, which can be written:

$$\boldsymbol{v_P} = (\mathrm{IO}[P], \mathrm{OS}[P, w = 1], \dots, \mathrm{OS}[P, w = 42], \mathrm{PB}[P, w = 1], \dots, \mathrm{PB}[P, w = 42])$$

where $\mathrm{IO}[P] \in [0, 3)$ determines the infrastructure option selected by partnership P according to the following mapping:

$$\text{Infrastructure option} = \begin{cases} \text{Canal expansion } if \ \mathrm{IO}[P] \in [0, 1) \\ \text{Groundwater bank } if \ \mathrm{IO}[P] \in [1,2) \\ \text{Both canal expansion \& groundwater bank } if \ \mathrm{IO}[P] \in [2,3) \end{cases}$$

and $\mathrm{OS}[P, w] \in [0, 1)$ determines the ownership share assigned to each water provider w. To map $\mathrm{OS}[P, w]$ to a valid set of ownership shares for simulation, any shares that are nonzero but less than 0.01 (i.e., 1%) are set to 0.01 to enforce the minimum ownership share requirement. Then all shares are reweighted to sum to 1. These two operations are iteratively repeated up to 5 times as needed. Finally, the shares are multiplied by 100 to convert them into percentages.

Finally, $\mathrm{PB}[P, w] \in [0, 1)$ is a binary switch variable that turns water provider w "on" or "off" as a partner. The mapping is defined such that the updated ownership share is written:

$$\mathrm{OS}'[P, w] = \begin{cases} 0 \ if \ \mathrm{PB}[P, w] \in [0, 0.5) \\ \mathrm{OS}[P, w] \ if \ \mathrm{PB}[P, w] \in [0.5, 1) \end{cases}$$

The binary switch variables allow the search algorithm to more easily explore a range of smaller partnership designs, which was found to speed up the search process in preliminary trials compared to alternative formulations without these switches. Similar formulations have proven effective for multiobjective metaheuristic optimization applied to other portfolio-style problems such as in finance[21].

References

1. Zeff, H. B. *et al.* California's food-energy-water system: An open source simulation model of adaptive surface and groundwater management in the Central Valley. *Environ. Model. Softw.* **141**, 105052–105052 (2021).
2. Lebedev, S. hmmlearn. https://hmmlearn.readthedocs.io/ (2015).

3. Nowak, K., Prairie, J., Rajagopalan, B. & Lall, U. A nonparametric stochastic approach for multisite disaggregation of annual to daily streamflow. *Water Resour. Res.* (2010) doi:10.1029/2009WR008530.

4. Taylor, K. E., Stouffer, R. J. & Meehl, G. A. An Overview of CMIP5 and the Experiment Design. *Bull. Am. Meteorol. Soc.* **93**, 485–498 (2012).

5. Brekke, L., Thrasher, B. L., Maurer, E. P. & Pruitt, T. *Downscaled CMIP3 and CMIP5 Climate Projections: Release of Downscaled CMIP5 Climate Projections, Comparison with Preceding Information, and Summary of User Needs*. (2013).

6. Liang, X., Lettenmaier, D. P., Wood, E. F. & Burges, S. J. A simple hydrologically based model of land surface water and energy fluxes for general circulation models. *J. Geophys. Res. Atmospheres* **99**, 14415–14428 (1994).

7. Cohen, J. S., Zeff, H. B. & Herman, J. D. Adaptation of Multiobjective Reservoir Operations to Snowpack Decline in the Western United States. *J. Water Resour. Plan. Manag.* **146**, 04020091–04020091 (2020).

8. Gent, P. R. *et al.* The Community Climate System Model Version 4. *J. Clim.* **24**, 4973–4991 (2011).

9. Voldoire, A. *et al.* The CNRM-CM5.1 global climate model: description and basic evaluation. *Clim. Dyn.* **40**, 2091–2121 (2013).

10. Collier, M. A. *et al.* The CSIRO-Mk3.6.0 Atmosphere-Ocean GCM: participation in CMIP5 and data publication. in *Chan, F., Marinova, D. and Anderssen, R.S. (eds) MODSIM2011, 19th International Congress on Modelling and Simulation.* (Modelling and Simulation Society of Australia and New Zealand (MSSANZ), Inc., 2011). doi:10.36334/modsim.2011.F5.collier.

11. Griffies, S. M. *et al.* The GFDL CM3 Coupled Climate Model: Characteristics of the Ocean and Sea Ice Simulations. *J. Clim.* **24**, 3520–3544 (2011).

12. Dunne, J. P. *et al.* GFDL's ESM2 Global Coupled Climate–Carbon Earth System Models. Part I: Physical Formulation and Baseline Simulation Characteristics. *J. Clim.* **25**, 6646–6665 (2012).

13. Martin, G. M. *et al.* The HadGEM2 family of Met Office Unified Model climate configurations. *Geosci. Model Dev.* **4**, 723–757 (2011).

14. Volodin, E. M., Dianskii, N. A. & Gusev, A. V. Simulating present-day climate with the INMCM4.0 coupled model of the atmospheric and oceanic general circulations. *Izv. Atmospheric Ocean. Phys.* **46**, 414–431 (2010).

15. Dufresne, J.-L. *et al.* Climate change projections using the IPSL-CM5 Earth System Model: from CMIP3 to CMIP5. *Clim. Dyn.* **40**, 2123–2165 (2013).

16. Watanabe, M. *et al.* Improved Climate Simulation by MIROC5: Mean States, Variability, and Climate Sensitivity. *J. Clim.* **23**, 6312–6335 (2010).

17. McPhail, C. *et al.* Robustness Metrics: How Are They Calculated, When Should They Be Used and Why Do They Give Different Results? *Earths Future* **6**, 169–191 (2018).

18. Herman, J. D., Zeff, H. B., Reed, P. M. & Characklis, G. W. Beyond optimality: Multistakeholder robustness tradeoffs for regional water portfolio planning under deep uncertainty. *Water Resour. Res.* **50**, 7692–7713 (2014).

19. Quinn, J. D., Reed, P. M., Giuliani, M. & Castelletti, A. Rival framings: A framework for discovering how problem formulation uncertainties shape risk management trade-offs in water resources systems. *Water Resour. Res.* **53**, 7208–7233 (2017).

20. Coello Coello, C. A., Lamont, G. B. & Van Veldhuizen, D. A. *Evolutionary Algorithms for Solving Multi-Objective Problems*. (Springer Science+Business Media, LLC, New York, NY, 2007). doi:10.1046/j.1365-2672.2000.00969.x.

21. Ponsich, A., Jaimes, A. L. & Coello Coello, C. A. A survey on multiobjective evolutionary algorithms for the solution of the portfolio optimization problem and other finance and economics applications. *IEEE Trans. Evol. Comput.* **17**, 321–344 (2013).
